# Phylogenetic and codon usage analysis of atypical porcine pestivirus (APPV)

**DOI:** 10.1080/21505594.2020.1790282

**Published:** 2020-07-29

**Authors:** Shuonan Pan, Chunxiao Mou, Huiguang Wu, Zhenhai Chen

**Affiliations:** a College of Veterinary Medicine, Yangzhou University, Yangzhou, Jiangsu, People’s Republic of China; b Institute of Comparative Medicine, Yangzhou University, Yangzhou, Jiangsu, People’s Republic of China; c Jiangsu Co-Innovation Center for Prevention and Control of Important Animal Infectious Diseases and Zoonoses, Yangzhou University, Yangzhou, Jiangsu, People’s Republic of China; d Joint International Research Laboratory of Agriculture and Agri-Product Safety, the Ministry of Education of China, Yangzhou University, Yangzhou, Jiangsu, People’s Republic of China

**Keywords:** Atypical porcine pestivirus, phylogenetics, codon usage, mutation pressure, selection pressure

## Abstract

Atypical porcine pestivirus (APPV) has been identified as the main causative agent for congenital tremor (CT) type A-II in piglets, which is threatening the health of the global swine herd. However, the evolution of APPV remains largely unknown. In this study, phylogenetic analysis showed that APPV could be divided into three phylogroups (I, II, and III). Phylogroups I and II included viral strains from China, while phylogroup III contained strains from Europe, North America, and Asia. Phylogroups I and II are tentatively thought to be of Chinese origin. Next, compositional property analysis revealed that a high frequency of nucleotide A and A-end codons was used in the APPV genome. Intriguingly, the analysis of preferred codons revealed that the AGA[Arg] and AGG[Arg] were overrepresented. Dinucleotide CC was found to be overrepresented, and dinucleotide CG was underrepresented. Furthermore, it was found that the weak codon usage bias of APPV was mainly dominated by selection pressures versus mutational forces. The codon adaptation index (CAI), relative codon deoptimization index (RCDI), and similarity index (SiD) analyses showed that the codon usage patterns of phylogroup II and III were more similar to the one of a pig than phylogroup I, suggesting that phylogroup II and III may be more adaptive to pigs. Overall, this study provides insights into APPV evolution through phylogeny and codon usage pattern analysis.

## Introduction

As an emerging novel swine virus, atypical porcine pestivirus (APPV) is a member of the genus *Pestivirus* within the family *Flaviviridae* [1]. Recently, APPV has been classified as *Pestivirus K* by the International Committee on Taxonomy of Viruses (ICTV) [2]. It is now regarded as the main causative agent for congenital tremor (CT) type A-II in piglets [3]. APPV was first identified in the USA using metagenomic sequencing in 2015 and has been reported in countries including Germany, Netherland, China, Spain, Brazil, South Korea, Canada, and Hungary, where it threatens the health of the global swine herd [1,3,4]. The virus has also been found in the serum samples of wild boars in Germany, implying that the wild boars may be an APPV reservoir worthy of epidemiological investigation [5].

APPV is an enveloped, single-stranded, and positive-sense RNA virus with a length of about 11–12 kb. The virus genome contains an open reading frame (ORF) encoding a polyprotein consisted of 3635 amino acids that were putatively processed into four structural proteins (C, E^rns^, E1, and E2) and eight non-structural proteins (N^pro^, P7, NS2, NS3, NS4A, NS4B, NS5A, and NS5B) [1]. APPV is highly variable in the virus genome among diverse strains, which undoubtedly poses several challenges for the development of diagnostic tests and vaccines, as well as virus research [4,6]. Thus far, the origin and evolution of APPV remain largely unclear, though a preliminary phylogeny exists including a number of APPV clusters with high variability.

For most organisms, the preference for specific codon usage (referred to as codon usage bias) is an important indicator of biological evolution. The evolution of codon bias is a complex process associated with several factors like natural selection, mutation pressure, genetic drift, and GC content [7–9]. During the evolutionary process, the genome may experience a diversification of codon usage/bias which leads to the changes in the efficiency of gene expression and protein production [10]. Codon usage patterns can therefore provide important information for investigating the evolution, host adaptation, and factors driving codon usage bias. The extent of codon usage bias between the virus and its hosts has been experimentally suggested to affect viral survival, replicative fitness, virulence, and protein synthesis [11–13].

Some pestiviruses have a relatively broad range of hosts. For example, bovine viral diarrhea virus (BVDV) and border disease virus (BDV) infect sheep, cattle, and pigs. In contrast, APPV and classical swine fever virus (CSFV) are only found in domestic pigs and wild boars. The differences in codon usage patterns observed among BVDV, CSFV, and BDV might reflect the relatively restricted host range used by these three viruses and might indicate a distinct evolutionary process for each virus [14–18]. These findings reveal the diversity of pestivirus evolution. Now that the number of newly discovered APPV isolates has increased significantly, a comprehensive genome analysis of APPV is warranted. In the present study, we attempted to elucidate the phylogenetic relationship of APPV strains using maximum likelihood estimation and Bayesian inference, and employed a broad range of methods to investigate the key factors responsible for the codon usage bias of APPV.

## Materials and methods

### Data analysis

All APPV sequences were collected from the GenBank database of the National Center for Biotechnology Information (NCBI) until November 2019. The detailed sequence information (accession number, strain name, location, and isolation year) for 61 complete genomic sequences of APPV is found in the supplementary material (Supplementary Table 1).

### Recombination detection and phylogenetic analysis

Potential recombination events in coding DNA sequences (CDSs) of APPV strains were identified using the recombination detecting program RDP4 (version 4.97) [19] with the default settings. Recombination analysis of the aligned sequences was performed with default configuration using seven different recombination detection algorithms viz. RDP, GENECONV, Chimeara, MaxChi, BootScan, 3Seq, and SiSca. A Bonferroni-corrected *P*-value cutoff of 0.05 was applied throughout the analysis. To avoid false-positive results, only recombination events supported by at least four different methods were considered. The remaining sequences were subject to recombination detection again with at least four different methods until there was no recombination signal.

The general time-reversible (GTR) model with gamma-distributed evolutionary rates (G) and invariable sites (I) (GTR+G + I) was chosen as the best fitting model based on Akaike information criterion (AIC) using jModelTest2 (version 2.1.10) [20]. Phylogenetic trees were reconstructed by maximum likelihood (ML) using RAxML (version 8.2.12) [21], and by Bayesian inference (BI) using MrBayes (version 3.2.7a) [22]. The robustness of clusters identified by ML was estimated by 1,000 bootstrap replicates. For BI, two chains ran for 1,000,000 generations with the first 25% set as burn-in. The phylogenetic trees were viewed in Figtree (version 1.4.4) (http://tree.bio.ed.ac.uk/software/figtree/).

### Pairwise genetic distance calculations

The pairwise genetic distances between three phylogroups were calculated using the DIVEIN [23] software with the GTR model of nucleotide substitutions and a gamma distribution with 4 parameters. The model was chosen using jModelTest2 (version 2.1.10) [20].

### Compositional and principal parameters analysis

The compositional characteristics of the APPV complete coding sequences were calculated and five nonsynonymous codons, ATG, TGG, and three termination codons were excluded from the analysis. The frequencies of mononucleotides (A, C, U, and G), GC contents at the first (GC1s), second (GC2s), third (GC3s) codon positions, and mean of GC1 and GC2 (GC12s) were computed by the seqinr package (version 3.6–1) of R (version 3.6.2) [24,25]. The frequencies of A, T, C, and G at the third positions (A3%, T3%, G3%, C3%) in the synonymous codons were estimated in Codon W software (version 1.4.2) developed by J. Peden (http://codonw.sourceforge.net/).

### Relative synonymous codon usage

Relative synonymous codon usage (RSCU) represents the ratio of the actual value to the expected value of the special codon in the synonymous codon [26], regardless of the effect of nucleotide composition and sequence length. The RSCU value was estimated as follows [27]:

(1) $$RSCU=\frac{g_{ij}}{\sum_{j}^{ni}g_{ij}}n_{i}$$

In the equation, *g_ij_* is the observed number of the *i_th_* codon for the *j_th_* amino acid, which has *n_i_* kinds of alternative synonymous codons estimated using the seqinr package (version 3.6–1) of R (version 3.6.2) [24,25]. The RSCU value = 1.0 indicates no codon usage bias. A RSCU value > 1.0 represents positive bias; however, a RSCU value < 1.0 represents negative bias. In addition, the value > 1.6 indicates “over-represented”, while < 0.6 indicates “underrepresented” [28].

### Principle component analysis

Principal component analysis (PCA) is a multivariate statistical method. As a main unsupervised linear transformation technique, PCA is widely used to feature extraction and dimensionality reduction [29]. In this study, each dimension represents a relative synonymous codon usage (RSCU) value of sense codon except for ATG, TGG, and three stop codons. A matrix containing 59 RSCU values per sequence was constructed for PCA and transformed into several major axes. PCA was performed using the factoextra package (version 1.0.6) of R (version 3.6.2) [25,30].

### Relative dinucleotide abundance analysis

The relative dinucleotide abundances representing the frequencies of 16 dinucleotides in codon usage pattern were computed using the following formula [31]:

(2) $$P_{xy}=\frac{f_{xy}}{f_{x}f_{y}}$$

In the formula, *f_x_* and *f_y_* stand for the frequency of nucleotide X and nucleotide Y, respectively. *f_x_f_y_* represents the expected frequency of dinucleotide XY, and *f_xy_* represents the estimated frequency of dinucleotide XY. *P_xy_* > 1.23 indicates that dinucleotide is overrepresented, while *P_xy_* < 0.78 indicates that dinucleotide is underrepresented. Meanwhile, the extremes of dinucleotide relative abundances can be distinguished as follows [32]: extremely overrepresented (*P_xy_* ≥ 1.50), very overrepresented (1.30 ≤ *P_xy_* < 1.50), significantly overrepresented (1.23 ≤ *P_xy_* < 1.30), marginally overrepresented (1.20 ≤ *P_xy_* < 1.23), extremely underrepresented (*P_x_* ≤ 0.50), very underrepresented (0.50 ≤ *P_xy_* < 0.70), significantly underrepresented (0.70 ≤ *P_xy_* < 0.78), and marginally underrepresented (0.78 ≤ *P_xy_* < 0.81).

### Effective number of codons analysis

An effective number of codon (ENC) analysis reflects the deviation of codon from random selection. The ENC value ranges from 20 to 61 [33]. The closer the value is to 21, the higher the codon bias is; the closer the value is to 60, the lower the codon bias is [33,34]. Notably, the ENC value is less than or equal to 45, which indicates a strong codon usage bias. The ENC value was calculated using the following formula [33]:

(3) $$ENC=2+\frac{9}{{\overset{ˉ}{F}}_{2}}+\frac{1}{{\overset{ˉ}{F}}_{3}}+\frac{5}{{\overset{ˉ}{F}}_{4}}+\frac{3}{{\overset{ˉ}{F}}_{6}}$$

where the *F_i_* (*i* = 2, 3, 4, 6) represents the average *F_i_* in the *i*-fold degenerate amino acid family. The *F_i_* value was calculated as follows [33]:

(4) $${\overset{ˉ}{F}}_{i}=\frac{n\sum_{j=1}^{i}{\left(\frac{n_{j}}{n}\right)}^{2}-1}{n-1}$$

where *n* represents the total number of observed codons for that amino acid; *n_j_* represents the total number of observed *j_th_* codon for that amino acid. The ENC values were computed using the cordon package (version 1.4.0) of R (version 3.6.2) [25,35].

### ENC-plot analysis

ENC-plot denotes the relationship between GC3 values and ENC values, which represents the factors influencing the codon usage bias (i.e., mutation pressure) [33]. If the expected ENC values lie on the standard curve, it indicates that the codon usage is only influenced by mutation pressure. When the codon usage bias is restricted by other factors (i.e., natural pressure), the point will fall below the theoretical curve. The expected ENC value was computed using the following formula:

(5) $$ENC_{expected}=2+s+\frac{29}{s^{2}+{\left(1-s\right)}^{2}}$$

where *s* represents the frequency of G or C at the third position of synonymous codons.

### Parity rule 2 analysis

Parity rule 2 (PR2) analysis was used to estimate the effect of natural selection and mutation pressure on the codon usage. The ordinate represents the [*A3/(A3+ U3)*] value while the abscissa represents the $\left[G3/\left(G3+C3\right)\right]$. The origin point is 0.5 (x = 0.5 and y = 0.5), which indicates that A = T and G = C. Points lying on the origin indicates no deviation between the selectivity and mutation event.

### Neutrality analysis

Neutrality analysis represents the ratio of GC3s to GC12s, and it is commonly used to investigate the dominant factor (natural selection or mutation pressure) affecting the codon usage bias [8]. In neutrality analysis, if the coefficients of GC3s are statistically significant and close to 1, mutation pressure is regarded as the main force shaping codon usage. The closer the slope is to 0, the less the effect of mutation pressure on codon usage. The slope = 0 indicates that codon usage bias is totally shaped by natural selection [8]. The linear relationship between GC3-variable and GC12-variable was estimated by using R (version 3.6.2) [25].

### Codon adaptation index analysis

The codon adaptation index (CAI) is a quantitative value that is used to estimate the adaptiveness of a gene toward the codons of highly expressed genes [36]. The values of the CAI range from 0 to 1. The sequence with the higher CAI value is thought to be the preferred adaptiveness. The CAI values for all APPV genes were calculated using CAIcal [37]. The reference datasets of synonymous codon usage patterns of the pig (*Sus scrofa*) were downloaded from the Codon and Codon Pair Usage Tables (CoCoPUTs) database [38] updated in January 2020.

### Relative codon deoptimization index analysis

The relative codon deoptimization index (RCDI) is used to compare the similarities in codon usage between gene and reference genomes [13]. If the codon usage of the pathogen is similar to the one of the host, and the RCDI value is close to 1, it will be regarded as a higher translation rate [39]. RCDI values were calculated using CAIcal [37].

### Similarity index analysis

The similarity index (SiD) is an indicator to evaluate the influence of host codon usage on pathogen codon usage [40]. SiD is calculated using the following equation [41]:

(6) $$R\left(A,B\right)=\frac{\sum_{i=1}^{59}a_{i}\times b_{i}}{\sqrt{\sum_{i=1}^{59}a_{i}^{2}\times \sum_{i=1}^{59}b_{i}^{2}}}$$

(7) $$D\left(A,B\right)=\frac{1-R\left(A,B\right)}{2}$$

where *a_i_* means the RSCU value for a specific synonymous codon for the pathogen coding sequence; *b_i_* is the RSCU value for the same codon of the host. *D(A, B)* means the effect of the overall codon usage of the host on that of pathogen. The higher the SiD value, the greater the host influence is on pathogen codon usage.

### Statistical analysis

Because the values of CAI, SiD, and RCDI were not strictly normally distributed and the phylogroups had unequal variances, nonparametric tests were used. Non-parametric Kruskal–Wallis test followed by Bonferroni-corrected Dunn’s multiple comparison test was separately used to investigate any statistically significant differences of CAI, SiD, and RCDI obtained in the different phylogroups. Data analysis package dunn.test (version 1.3.5) [42] of R (version 3.6.2) [25] was used to perform this statistical analysis. A *P*-value < 0.05 was used as the cutoff criterion.

## Results

### Recombination and phylogenetic analysis

A total of 61 APPV complete CDSs were obtained from GenBank in November 2019. To avoid the potential effects of recombination on the topology of the phylogenetic tree, the APPV CDSs were analyzed using the recombination detecting program RDP4 (version 4.97) [19]. Eight of 61 APPV CDSs were found to have potential recombination signals. After removing the recombinant APPV CDSs, the remaining 53 sequences were used for further analysis (Supplementary Table 1).

**Figure 1.**
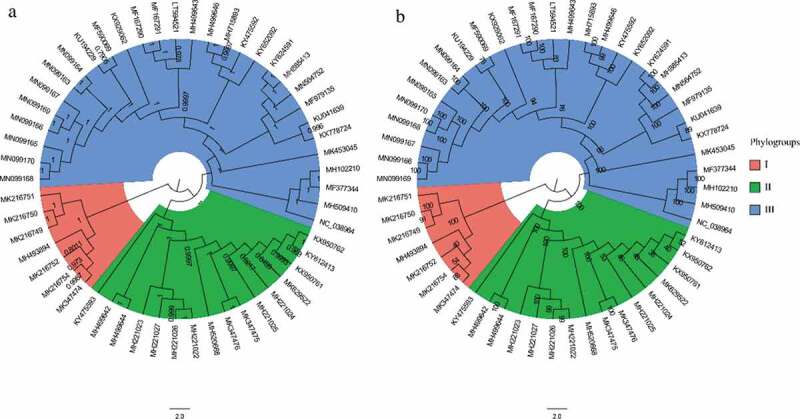
Phylogenetic trees of 53 complete genomes of APPV. (a) Bayesian Inference tree of the APPV genomes reconstructed by MrBayes. Posterior probability values are shown at each node. (b) Maximum likelihood tree of the APPV genomes reconstructed by RAxML. Bootstrap support values are indicated on the tree as a percentage of 1000 replicates. The colored circular sectors indicate the three phylogroups among APPV strains. Phylogroups I, II, and III are represented in orange, green, and blue, respectively.

To explore the phylogenetic relationship among APPV strains, we reconstructed the phylogenetic trees using ML and BI methods. In general, both ML and BI trees displayed the same topology (Figure 1). All known APPV strains could be placed into three well-supported phylogroups (I, II, and III). The phylogroups I and II comprised only the virus strains from China, while the phylogroup III included strains isolated from Europe, North America, and Asia. The genetic distances between phylogroups I and II, phylogroups I and III, and phylogroups II and III were 0.2451 ± 0.0002, 0.2350 ± 0.0003, and 0.2063 ± 0.0001, respectively (Supplementary Table 5).

Our results showed that the phylogroups I and II of APPV exhibited a phylogenetic pattern related to geographic distribution. Notably, the APPV strains of phylogroup I sampled from China exhibited the greatest genetic distance and fell at the basal positions of the phylogenetic trees with respect to the phylogroups II and III. These results indicated that APPV stains in the phylogroups I and II may have originated from China, though it needs to be identified on a far larger sample of taxa. The three phylogroups of APPV strains were then used to explore codon usage bias.

### PCA

**Figure 2.**
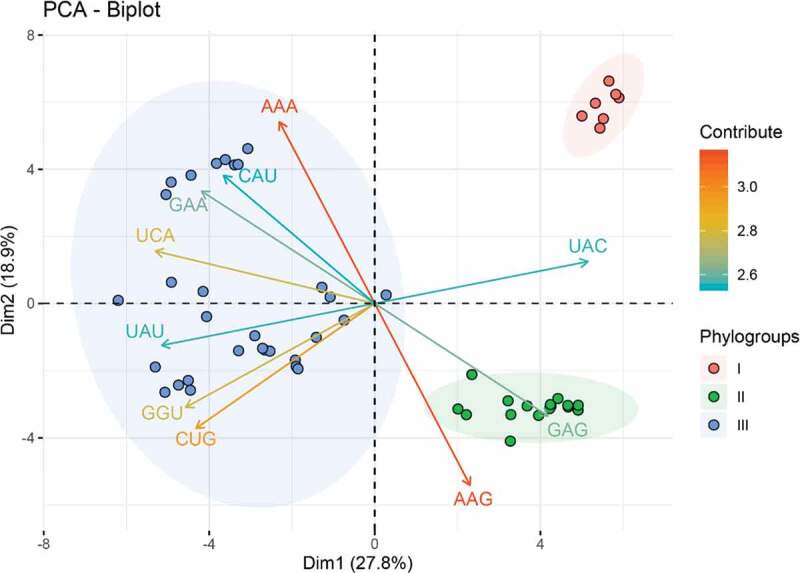
Principal component analysis (PCA) biplot diagram showing similarity and variation in codon usage pattern of APPV genomes. A PCA biplot is used for simultaneously displaying the genome CDSs of APPV and vectors of variables’ (lines) projection to the first two principal components. Biplot conducts on ten among most representative variables (codons) from all APPV strains. The direction and length of the arrows indicate how each codon contributes to the first two components in the biplot. Principal component 1 (Dimension 1) represents 27.8% of variation and principal component 2 (Dimension 2) represents 18.9% variation. Phylogroup I, II, and III are depicted using orange, green, and blue, respectively.

Using the RSCU values as descriptor variables, an unsupervised classification method PCA was performed to explore the codon usage features and evolutionary trends of APPV. The first and second principal components accounted for 27.8% and 18.9% of the total synonymous codon usage variation (Figure 2 and Supplementary Figure 1), respectively. From the PCA plot, we observed three distinctly separate groups corresponding to three phylogroups divided by phylogenetic relationships. The overall codon usage pattern of the phylogroup I isolated from China is dissimilar to those of phylogroup II from China and phylogroup III from Europe, North America, and Asia. The results of codon usage patterns from PCA showed a geographical distribution of the three APPV phylogroups, suggesting the geographical factor may influence APPV evolution potentially.

### Nucleotide A are the most frequent in APPV coding sequences

The mean composition of nucleotide A (0.317 ± 0.001) was the highest, followed by G (0.253 ± 0.001), U (0.223 ± 0.001), and C (0.208 ± 0.001) (Table 1 and Supplementary Table 2). The nucleotides at the third positions of synonymous codons showed the similar composition patterns: the mean content of A3s (0.39 ± 0.005) was higher than C3s (0.308 ± 0.004), G3s (0.302 ± 0.006), and U3s (0.264 ± 0.006). The mean contents of GCs and GC3s were 0.46 ± 0.002 and 0.495 ± 0.007, respectively. The mean compositions of GC1s (0.5 ± 0.002) and GC3s (0.495 ± 0.007) were the highest, followed by GC12s (0.443 ± 0.001) and GC2s (0.386 ± 0.002). The ENC values of APPV strains were 54.658 ± 0.091 (phylogroup I), 54.887 ± 0.091 (phylogroup II), and 54.843 ± 0.308 (phylogroup III), respectively, revealing a low codon usage bias in the APPV ORF sequences. These analyses indicated that the nucleotide A is highly presented in APPV coding sequences, and nucleotides at the third position of codons were GC-rich in the APPV coding sequences.

**Table 1.** Nucleotide composition of APPV complete genomes.

Categories	Phylogroup I	Phylogroup II	Phylogroup III	All
A	0.317 ± 0	0.315 ± 0.001	0.317 ± 0.001	0.317 ± 0.001
C	0.208 ± 0.001	0.208 ± 0.001	0.207 ± 0.001	0.208 ± 0.001
G	0.253 ± 0.001	0.254 ± 0	0.252 ± 0.001	0.253 ± 0.001
U	0.222 ± 0.001	0.222 ± 0.001	0.224 ± 0.001	0.223 ± 0.001
GC	0.461 ± 0.001	0.462 ± 0.001	0.459 ± 0.002	0.46 ± 0.002
GC1s	0.497 ± 0.001	0.501 ± 0.001	0.5 ± 0.002	0.5 ± 0.002
GC2s	0.384 ± 0.001	0.385 ± 0.001	0.387 ± 0.002	0.386 ± 0.002
GC12s	0.441 ± 0	0.443 ± 0.001	0.443 ± 0.001	0.443 ± 0.001
GC3s	0.501 ± 0.001	0.501 ± 0.003	0.49 ± 0.005	0.495 ± 0.007
U3s	0.253 ± 0.001	0.262 ± 0.003	0.268 ± 0.004	0.264 ± 0.006
C3s	0.311 ± 0.002	0.31 ± 0.002	0.305 ± 0.004	0.308 ± 0.004
A3s	0.393 ± 0.002	0.385 ± 0.003	0.392 ± 0.004	0.39 ± 0.005
G3s	0.306 ± 0.002	0.307 ± 0.003	0.298 ± 0.004	0.302 ± 0.006
ENC	54.658 ± 0.091	54.887 ± 0.142	54.843 ± 0.308	54.832 ± 0.254

A, U, C, and G represent the content of A, U, C, and G in the APPV sequences, respectively. GC1s, GC2s, and GC3s represent the GC content at the first, second and third codon positions, respectively. GC12s represents the mean value of GC1s and GC2s. A3s, U3s, C3s, and G3s represent the content of A, U, C, and G at the third codon positions.

### Unique relative synonymous codon usage of APPV

All three APPV phylogroups shared 11 preferred synonymous codons, including GCA[Ala], GAC[Asp], GAA[Glu], UUC[Phe], GGG[Gly], AUA[Ile], AAA[Lys], CCA[Pro], CAA[Gln], ACC[Thr], and GUG[Val] (Supplementary Table 3). The numbers of A/U-ended preferred codons in the phylogroups I, II, and III were 10, 8, and 11, respectively. Surprisingly, among 59 codons, only two (AGA and AGG) of six synonymous codons for Arg were over-represented (RSCU values >1.6) in the three phylogroups, while the remaining four codons (CGA, CGC, CGG, and CGU) were under-represented (RSCU values <0.6), with the exception of the codon CGG (0.606 ± 0.022) in the phylogroup I. In addition, four codons (GCG[Ala], UCG[Ser], ACG[Thr], and GUU[Val]) were under-represented (RSCU values <0.6) in the three APPV phylogroups. In conclusion, RSCU analysis showed that the coding sequences of APPV have a unique codon usage pattern which was influenced by evolution to a certain degree.

### Relative dinucleotide abundance of APPV

**Figure 3.**
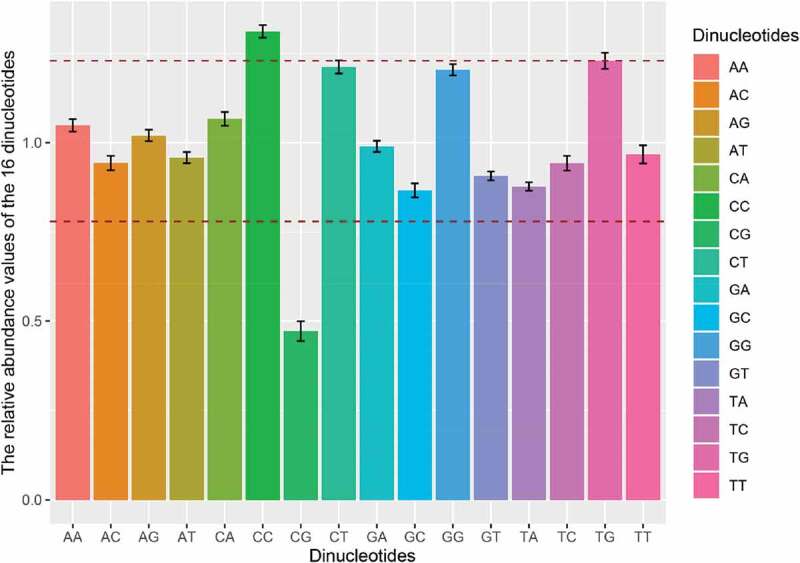
Dinucleotide abundance of the complete CDS of APPV. The different colors represent the different dinucleotides. Dinucleotides are regarded as underrepresented or overrepresented if the relative abundance values are below 0.78 or over 1.23 (dashed lines), respectively.

Considering the relative abundance of dinucleotides affecting the pattern of codon usage in RNA viruses, we calculated the relative abundance of 16 dinucleotides for APPV ORF sequences. None of the values was consistent with the theoretical value (equal to 1.0), and all the relative dinucleotide abundance values were at different usage frequencies (Figure 3 and Supplementary Table 4). Dinucleotides CC and TG were overrepresented (*P_xy_* ≥ 1.23), while dinucleotide CG was extremely underrepresented (*P_xy_* ≤ 0.50). The dinucleotide CT (*P_xy_* = 1.213 ± 0.018) and GG (*P_xy_* = 1.205 ± 0.016) were marginally over-represented. Especially, dinucleotide CA (*P_xy_* = 1.249 ± 0.01) was overrepresented in the genome CDSs of APPV phylogroup II. These results indicated that APPV had a unique dinucleotide usage pattern.

### The effect of mutation pressure and natural selection on codon usage bias

**Figure 4.**
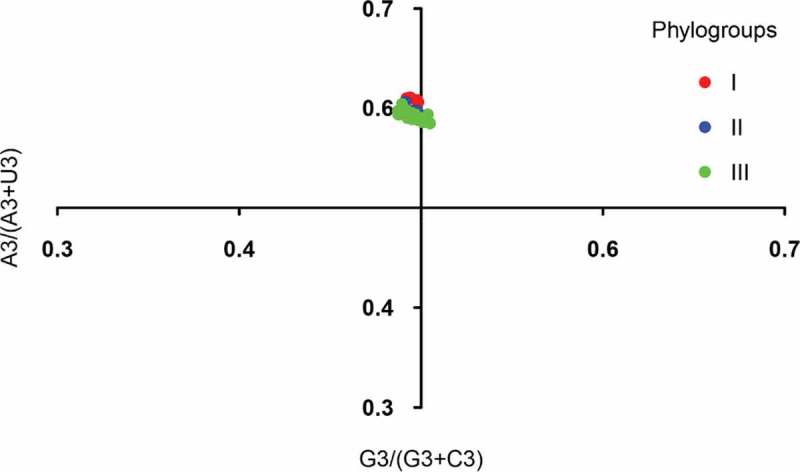
PR2 plot analysis of the APPV complete coding genomes. Phylogroups I, II, and III are represented in orange, green, and blue, respectively.

**Figure 5.**
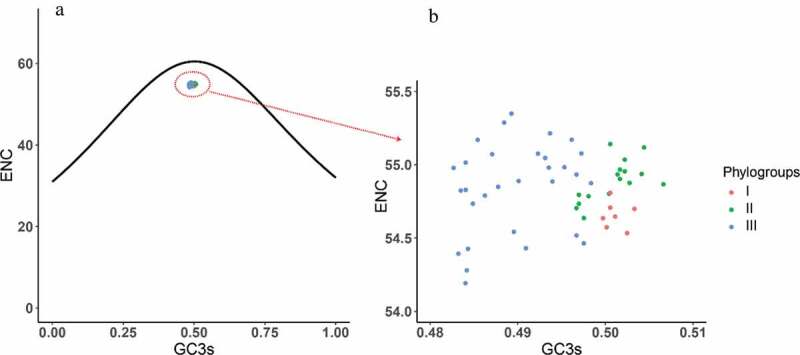
ENC plot analysis of the complete coding genomes of APPV. The ENC diagram shows relationship between ENC value and GC content at the third position (GC3s). The curve in the plot represents the expected ENC value for all GC3 compositions. Phylogroups I, II, and III are represented in orange, green, and blue, respectively.

To explore the factors that influence the codon usage pattern, PR2 bias analysis, ENC-plot analysis, and neutrality analyses of different genotypes were employed. Most of the points in the PR2 plot fell near 0.7 of the vertical axes, implying that A3 was used more frequently than U3 (Figure 4). These data provide evidence that there are forces driving the formation of the codon usage pattern of the APPV. All the points in ENC-plot distinctly fell below the expected curve regardless of phylogroups (Figure 5). The results of ENC-plot indicate that for all CDSs, selection pressure is the major force influencing the codon usage in APPV.

**Figure 6.**
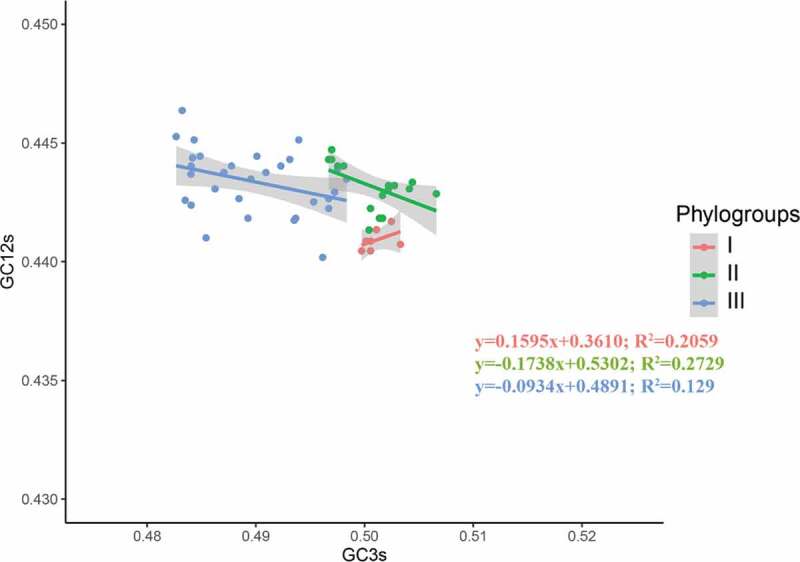
Neutrality analysis of the APPV complete coding genomes. The neutrality plot shows the correlation between GC content in synonymous positions (GC12s) and GC content in non-synonymous positions (GC3s). Phylogroups I, II, and III are represented in orange, green, and blue, respectively.

To investigate which factor played a major role in the codon usage pattern, neutrality analysis was carried out. A significant negative correlation between GC3 and GC12 was observed in the APPV phylogroup II (*y* = −0.1738*x*+0.5302; R^2^ = 0.2729; *P* < 0.05) (Figure 6), suggesting the natural selection contributes 82.62% of the action to model the codon usage pattern of the APPV phylogroup II. For phylogroups I and III, there was no correlation between GC3s and GC12s (*P* > 0.05), indicating that codon usage patterns of APPV phylogroups I and III were the result of natural selection.

### Comparison between the codon pattern of phylogroups toward its host

We compared the codon usage preference of APPV phylogroups in relation to the codon usage preference of pig using the analyses of CAI, SiD, and RCDI. For CAI, phylogroups II and III were significantly higher than phylogroup I (*P* < 0.05, Dunn’s test), and there was no statistical difference in the CAI between phylogroups II and III (*P* > 0.05, Dunn’s test) (Supplementary Figure 2). We found that SiD of phylogroup I was significantly higher than that of phylogroups II and III (*P* < 0.05, Dunn’s test), but did not find a significant difference between phylogroups II and III (*P* > 0.05, Dunn’s test) (Supplementary Figure 3). The differences in RCDI among three phylogroups of APPV were significant (*P* < 0.01, Kruskal–Wallis test); *post hoc* comparisons showed that the differences were significant between the phylogroups I and II, phylogroups I and III, and phylogroups II and III (*P* < 0.05, Dunn’s test) (Supplementary Figure 4). These results of CAI, SiD, and RCDI could be a reflection of the differences in codon usage of APPV phylogroups in relation to the pig.

## Discussion

In the present study, we comprehensively analyzed the phylogenetic relationship of APPV. Phylogenetic analysis demonstrated that all APPV strains were divided into three phylogroups (I, II, and III). One notable feature was that the strains of phylogroup I isolated from China occupied the basal position of the phylogenetic tree, and were distinct from strains of the two other phylogroups in the PCA plot. Our results tentatively support a Chinese origin for APPV strains of phylogroups I and II. However, it needs to be further assessed on more geographically diverse samples.

The composition of nucleotides is considered to be an important factor affecting codon usage. It is interesting to note that the percentages of nucleotide A and A-end codons are the highest in the APPV genomes. Consistent with previous findings in the BVDV [14] and CSFV [15], a high content of nucleotide A in the APPV CDSs might be a genomic characteristic of the genus *Pestivirus*.

The ENC values of each APPV genome were calculated to estimate the overall codon usage bias. The mean ENC values of APPV genomes (54.832 ± 0.254) were higher than BVDV (51.43 ± 0.46) [14], CSFV (51.85 ± 0.39) [15], and BDV (average 51.33 and range from 51.12 to 51.55), suggesting that the overall codon bias of APPV is considerably weaker. Considering that low codon usage bias may be useful for efficient replication with more codon selection options [43], our results showed that the low codon bias of APPV might facilitate its genome replication and transcription.

The preferentially A/T-ended codons of APPV were found inconsistent with those of BVDV [14] and CSFV [15], in which G/C-ended codons were preferentially used. Notably, two (AGA[Arg] and AGG[Arg]) over-represented codons and seven (GCG[Ala], UCG[Ser], ACG[Thr], CGU[Arg], CGC[Arg], CGA[Arg], and CGG[Arg]) under-represented codons were frequently presented in the genomes of APPV, BVDV [14], and CSFV [15], indicating a common genomic feature of codon usage patterns of pestiviruses. In conclusion, APPV has a common characteristic of genus *Pestivirus* as well as its own unique characteristic in the codon usage pattern.

Codon usage bias may also be affected by dinucleotide frequency. An imbalanced usage pattern of 16 dinucleotides was observed in the APPV genomes. In our study, we found that dinucleotide CC was over-enriched, while dinucleotide CG was under-enriched in the genomes of APPV, which was consistent with CSFV [44]. Statistically, dinucleotide CG is underrepresented in most small viruses (lengths of <30 kb) [45]. However, the relatively high abundance of dinucleotide CC in the APPV genomes has more thermodynamic stacking energy resulting in a low transcription and replication efficiency compared to G/C-free dinucleotides. Indeed, APPV is not a highly virulent pathogen with subclinical features [46], which might relate to the low replication efficiency caused by the high content of dinucleotide CC.

PR2 bias, ENC-plot, and neutrality analyses showed natural selection dominates the codon usage bias of APPV. However, the results of this study are completely inconsistent with the analysis of CSFV, BVDV, and BDV, where mutational pressure may be the main factor in determining the codon usage bias [14–17]. These results indicate the evolutionary process of APPV is unique, differing from the CSFV, BVDV, and BDV.

The CAI, RCDI, and SiD are used to evaluate the expression level of pathogen proteins in the host, to make comparisons of codon usage in different organisms, and to evaluate viral adaptation to host. As intracellular parasites, viruses are totally dependent on the translation machinery of host for their propagation. Effective replication of the virus requires similar codon usage patterns between the virus and the host which shared the same amino acid/tRNA pool. In the present study, the phylogroup I had a relatively lower CAI value, and higher values of SiD and RCDI than the phylogroups II and III. These results demonstrated that the phylogroups II and III have a more similar codon usage pattern with pigs, which makes them more adaptive to host.

In summary, we firstly provided information about the phylogenetic relationship, codon usage pattern, factors affecting the codon usage, and host adaptation of APPV. Based on phylogenetic analysis and PCA, APPV strains are divided into three phylogroups (I, II, and III) with geographical characteristics, and phylogroups I and II are tentatively thought to be of Chinese origin. Nucleotide A and A-end codons are highly frequently presented in the genome CDSs of APPV, and unique patterns of synonymous codon usage and dinucleotide usage are identified. APPV has a weak codon usage bias, which was mainly affected by natural selection. Overall, these results will serve future APPV surveillance and basic research, and provide important insights into the understanding of APPV evolution.

## Supplementary Material

Supplemental Material

Supplemental Material
