# Supplementary material for: Phylogenetic and codon usage analysis of atypical porcine pestivirus (APPV)
Source: Virulence. 2020 Jul 29;11(1):916–26. doi: 10.1080/21505594.2020.1790282 (PMC7549985; doi:10.1080/21505594.2020.1790282)
Supplement: Supplemental Material [file KVIR_A_1790282_SM8597.docx]

Supplementary Table 1. The detailed information (accession number, strain name, isolated country, as well as collection date) of the complete coding genome of APPV.

| **Accession number** | **Strain name** | **Country** | **Recombinant** | | **Collection date** |
| --- | --- | --- | --- | --- | --- |
| MH493894 | GD-MH01-2018 | China | No | 2018.02 | |
| MK216751 | AH-SG-2018.01 | China | No | 2018.01 | |
| MK216749 | AH-GL-2017.04 | China | No | 2017.04 | |
| MK216750 | AH-GL-2018.01 | China | No | 2018.01 | |
| MK216752 | GD-HJ-2017.04 | China | No | 2017.04 | |
| MK216754 | GD-ZW-2017.10 | China | No | 2017.1 | |
| MK347474 | GD-LDCT1 | China | No | 2018 | |
| KY475593 | APPV-China/GD-SD/2016 | China | No | 2016.12 | |
| MH499642 | APPV-China/SWU-DY/2018 | China | No | 2018.03.25 | |
| MH499644 | APPV-China/SWU-MY/2018 | China | No | 2018.05.08 | |
| MH221023 | APPV-China/GD-HE/2016 | China | No | 2016.04 | |
| MH221027 | APPV-China/GD-ST/2016 | China | No | 2016.04 | |
| MH221026 | APPV-China/GD-SHT/2016 | China | No | 2016.04 | |
| MH221022 | APPV-China/GD-GL/2016 | China | No | 2016.04 | |
| MH520668 | GD-BH02-2018 | China | No | 2018.02 | |
| MK347475 | GD-YJHSEY2N | China | No | 2017 | |
| MK347476 | GD-YJHSEY3N | China | No | 2017 | |
| MH221025 | APPV-China/GD-SHM/2016 | China | No | 2016.04 | |
| MH221024 | APPV-China/GD-HG/2016 | China | No | 2016.04 | |
| MK629522 | CH-GD2017 | China | No | 2017.12.30 | |
| KX950761 | GD1 | China | No | 2016.07.20 | |
| KY612413 | GD3 | China | No | 2016.11.15 | |
| KX950762 | GD2 | China | No | 2016.08.03 | |
| NC_038964 | 000515 | USA | No | 2014.04.09 | |
| MH509410 | KU16-6 | South_Korea | No | 2016 | |
| MH102210 | GX04/2017 | China | No | 2017.11 | |
| MF377344 | HBtl1701 | China | No | 2017 | |
| MK453045 | GX02-2018 | China | No | 2018 | |
| KY652092 | APPV_GX-CH 2016 | China | No | 2016.12.16 | |
| KY475592 | APPV-China/GZ01/2016 | China | No | 2016.12 | |
| MH715893 | GX01-2018 | China | No | 2018 | |
| MH499646 | APPV-China/SWU-YB/2018 | China | No | 2018.05.18 | |
| KX778724 | AUT-2016_C | Austria | No | 2016 | |
| KU041639 | Bavaria S5/9 | Germany | No | 2015 | |
| MF979135 | KU16-2 | South_Korea | No | 2016 | |
| MH885413 | HN2018 | China | No | 2018.08 | |
| KY624591 | APPV_GD | China | No | 2016 | |
| MH499643 | APPV-China/SWU-KZ/2018 | China | No | 2018.04.06 | |
| LT594521 | APPV_GER_01 | Germany | No | beforoe2016.05.13 | |
| MF167290 | Ger-NRW_CT-59 | Germany | No | 2015 | |
| MF167291 | Ger-NRW_L277 | Germany | No | 2016 | |
| KX929062 | NL1 Farm1 | Netherlands | No | 2012.03 | |
| MF590069 | APPV/Pig-wt/USA/Minnesota-1/2016 | USA | No | 2016.09.15 | |
| KU194229 | ISDVDL2014016573 | USA | No | 2014.08 | |
| MN099163 | 8247 | Switzerland | No | 2006 | |
| MN099164 | 2086 | Switzerland | No | 2006 | |
| MN099165 | SK68-11 | Switzerland | No | 2011.07.08 | |
| MN099168 | 170575-1 | Switzerland | No | 2017.07.24 | |
| MN099170 | 180416 | Switzerland | No | 2018 | |
| MN099167 | 5620 | Switzerland | No | 2015 | |
| MN099166 | S13-0310 | Switzerland | No | 2013.03.11 | |
| MN099169 | 170711-1 | Switzerland | No | 2017.10.11 | |
| MN564752 | GX01-2019 | China | No | 2019 | |
| MF167292 | CN-CQ_11/39 | China | Yes | 2014 | |
| MH499645 | APPV-China/SWU-XC/2017 | China | Yes | 2017.11.26 | |
| MH499648 | APPV-China/SWU-QL/2018 | China | Yes | 2018.6.12 | |
| MH499647 | APPV-China/SWU-ZH/2017 | China | Yes | 2017.8.17 | |
| MK216753 | GD-LN-2017.04 | China | Yes | 2017.4 | |
| MG792803 | JX-JM01-2018A01 | China | Yes | 2016.3 | |
| MH493896 | GD-BZ01-2018 | China | Yes | 2018.2 | |
| MH493895 | GD-DH01-2018 | China | Yes | 2018.2 | |

Supplementary Table 2. The nucleotide composition and properties of APPV.

| Strain | A% | C% | G% | T% | G%+C% | GC1s | GC2s | GC12s | GC3s | T3s | C3s | A3s | G3s | ENC |
| --- | --- | --- | --- | --- | --- | --- | --- | --- | --- | --- | --- | --- | --- | --- |
| KU041639 | 0.316 | 0.209 | 0.252 | 0.222 | 0.462 | 0.501 | 0.387 | 0.444 | 0.498 | 0.264 | 0.313 | 0.386 | 0.302 | 54.464 |
| KU194229 | 0.317 | 0.207 | 0.252 | 0.224 | 0.459 | 0.499 | 0.384 | 0.442 | 0.494 | 0.264 | 0.310 | 0.392 | 0.298 | 55.215 |
| KX778724 | 0.316 | 0.207 | 0.253 | 0.223 | 0.461 | 0.500 | 0.386 | 0.443 | 0.497 | 0.264 | 0.309 | 0.387 | 0.305 | 54.518 |
| KX929062 | 0.316 | 0.204 | 0.253 | 0.227 | 0.457 | 0.500 | 0.388 | 0.444 | 0.484 | 0.277 | 0.295 | 0.390 | 0.301 | 55.015 |
| KX950761 | 0.314 | 0.209 | 0.255 | 0.222 | 0.464 | 0.501 | 0.386 | 0.443 | 0.504 | 0.262 | 0.311 | 0.380 | 0.312 | 55.118 |
| KX950762 | 0.315 | 0.208 | 0.254 | 0.222 | 0.463 | 0.501 | 0.385 | 0.443 | 0.502 | 0.262 | 0.311 | 0.382 | 0.309 | 54.956 |
| KY475592 | 0.316 | 0.207 | 0.252 | 0.225 | 0.459 | 0.499 | 0.384 | 0.442 | 0.494 | 0.270 | 0.307 | 0.387 | 0.301 | 54.981 |
| KY475593 | 0.316 | 0.210 | 0.254 | 0.220 | 0.464 | 0.499 | 0.387 | 0.443 | 0.507 | 0.252 | 0.318 | 0.388 | 0.307 | 54.867 |
| KY612413 | 0.314 | 0.209 | 0.255 | 0.222 | 0.463 | 0.501 | 0.385 | 0.443 | 0.504 | 0.262 | 0.312 | 0.380 | 0.310 | 54.937 |
| KY624591 | 0.318 | 0.207 | 0.252 | 0.224 | 0.458 | 0.503 | 0.388 | 0.445 | 0.484 | 0.272 | 0.300 | 0.394 | 0.297 | 54.427 |
| KY652092 | 0.315 | 0.208 | 0.253 | 0.224 | 0.461 | 0.500 | 0.386 | 0.443 | 0.497 | 0.267 | 0.311 | 0.384 | 0.302 | 55.077 |
| LT594521 | 0.316 | 0.209 | 0.253 | 0.222 | 0.461 | 0.503 | 0.387 | 0.445 | 0.494 | 0.267 | 0.309 | 0.389 | 0.299 | 54.886 |
| MF167290 | 0.315 | 0.207 | 0.254 | 0.224 | 0.460 | 0.501 | 0.388 | 0.444 | 0.492 | 0.272 | 0.303 | 0.385 | 0.304 | 55.076 |
| MF167291 | 0.316 | 0.207 | 0.253 | 0.224 | 0.461 | 0.501 | 0.388 | 0.444 | 0.493 | 0.270 | 0.305 | 0.386 | 0.303 | 55.046 |
| MF377344 | 0.319 | 0.206 | 0.250 | 0.225 | 0.456 | 0.496 | 0.386 | 0.441 | 0.485 | 0.269 | 0.305 | 0.397 | 0.293 | 55.170 |
| MF590069 | 0.317 | 0.207 | 0.251 | 0.224 | 0.458 | 0.500 | 0.388 | 0.444 | 0.487 | 0.270 | 0.307 | 0.394 | 0.292 | 55.072 |
| MF979135 | 0.318 | 0.208 | 0.251 | 0.223 | 0.459 | 0.502 | 0.390 | 0.446 | 0.483 | 0.269 | 0.305 | 0.398 | 0.289 | 54.393 |
| MH102210 | 0.317 | 0.207 | 0.251 | 0.225 | 0.458 | 0.499 | 0.386 | 0.443 | 0.488 | 0.267 | 0.306 | 0.395 | 0.296 | 55.288 |
| MH221022 | 0.316 | 0.208 | 0.254 | 0.222 | 0.462 | 0.502 | 0.386 | 0.444 | 0.497 | 0.263 | 0.308 | 0.389 | 0.304 | 54.734 |
| MH221023 | 0.315 | 0.208 | 0.254 | 0.223 | 0.462 | 0.503 | 0.386 | 0.445 | 0.497 | 0.264 | 0.309 | 0.388 | 0.303 | 54.795 |
| MH221024 | 0.315 | 0.208 | 0.254 | 0.223 | 0.462 | 0.500 | 0.385 | 0.442 | 0.501 | 0.264 | 0.310 | 0.383 | 0.308 | 55.141 |
| MH221025 | 0.315 | 0.208 | 0.254 | 0.223 | 0.462 | 0.501 | 0.384 | 0.443 | 0.502 | 0.263 | 0.310 | 0.383 | 0.309 | 54.967 |
| MH221026 | 0.316 | 0.208 | 0.254 | 0.222 | 0.462 | 0.503 | 0.386 | 0.444 | 0.497 | 0.263 | 0.309 | 0.389 | 0.303 | 54.705 |
| MH221027 | 0.316 | 0.208 | 0.254 | 0.222 | 0.462 | 0.503 | 0.385 | 0.444 | 0.498 | 0.262 | 0.309 | 0.389 | 0.304 | 54.786 |
| MH493894 | 0.317 | 0.208 | 0.254 | 0.222 | 0.461 | 0.497 | 0.385 | 0.441 | 0.501 | 0.253 | 0.310 | 0.393 | 0.307 | 54.647 |
| MH499642 | 0.316 | 0.208 | 0.254 | 0.222 | 0.462 | 0.502 | 0.386 | 0.444 | 0.498 | 0.263 | 0.307 | 0.389 | 0.305 | 54.637 |
| MH499643 | 0.318 | 0.207 | 0.250 | 0.224 | 0.458 | 0.501 | 0.390 | 0.445 | 0.483 | 0.272 | 0.301 | 0.397 | 0.292 | 54.979 |
| MH499644 | 0.315 | 0.208 | 0.255 | 0.222 | 0.463 | 0.501 | 0.386 | 0.443 | 0.503 | 0.260 | 0.311 | 0.385 | 0.308 | 54.877 |
| MH499646 | 0.317 | 0.208 | 0.252 | 0.223 | 0.460 | 0.499 | 0.386 | 0.443 | 0.495 | 0.266 | 0.311 | 0.388 | 0.299 | 54.984 |
| MH509410 | 0.317 | 0.206 | 0.251 | 0.225 | 0.458 | 0.498 | 0.386 | 0.442 | 0.489 | 0.269 | 0.305 | 0.393 | 0.298 | 55.349 |
| MH520668 | 0.315 | 0.207 | 0.254 | 0.224 | 0.461 | 0.499 | 0.384 | 0.441 | 0.500 | 0.264 | 0.310 | 0.384 | 0.308 | 54.804 |
| MH715893 | 0.316 | 0.208 | 0.253 | 0.224 | 0.460 | 0.499 | 0.386 | 0.442 | 0.497 | 0.266 | 0.311 | 0.386 | 0.302 | 54.934 |
| MH885413 | 0.319 | 0.208 | 0.250 | 0.223 | 0.458 | 0.501 | 0.388 | 0.444 | 0.484 | 0.270 | 0.304 | 0.398 | 0.292 | 54.280 |
| MK216749 | 0.317 | 0.207 | 0.254 | 0.222 | 0.461 | 0.498 | 0.384 | 0.441 | 0.501 | 0.255 | 0.310 | 0.392 | 0.308 | 54.708 |
| MK216750 | 0.317 | 0.207 | 0.254 | 0.223 | 0.460 | 0.497 | 0.384 | 0.440 | 0.500 | 0.255 | 0.309 | 0.393 | 0.307 | 54.636 |
| MK216751 | 0.317 | 0.208 | 0.254 | 0.222 | 0.462 | 0.497 | 0.384 | 0.441 | 0.503 | 0.253 | 0.312 | 0.391 | 0.309 | 54.699 |
| MK216752 | 0.317 | 0.208 | 0.253 | 0.222 | 0.460 | 0.496 | 0.384 | 0.440 | 0.501 | 0.253 | 0.314 | 0.395 | 0.304 | 54.809 |
| MK216754 | 0.317 | 0.208 | 0.253 | 0.222 | 0.461 | 0.498 | 0.384 | 0.441 | 0.500 | 0.252 | 0.312 | 0.396 | 0.305 | 54.574 |
| MK347474 | 0.317 | 0.209 | 0.253 | 0.221 | 0.462 | 0.498 | 0.385 | 0.442 | 0.502 | 0.251 | 0.313 | 0.394 | 0.306 | 54.534 |
| MK347475 | 0.315 | 0.208 | 0.254 | 0.223 | 0.462 | 0.500 | 0.383 | 0.442 | 0.502 | 0.262 | 0.311 | 0.384 | 0.308 | 54.904 |
| MK347476 | 0.315 | 0.208 | 0.254 | 0.223 | 0.462 | 0.500 | 0.384 | 0.442 | 0.501 | 0.262 | 0.310 | 0.384 | 0.308 | 54.934 |
| MK453045 | 0.317 | 0.209 | 0.253 | 0.221 | 0.462 | 0.501 | 0.386 | 0.443 | 0.498 | 0.257 | 0.313 | 0.393 | 0.300 | 54.875 |
| MK629522 | 0.315 | 0.208 | 0.254 | 0.223 | 0.463 | 0.501 | 0.385 | 0.443 | 0.502 | 0.262 | 0.311 | 0.383 | 0.309 | 55.035 |
| MN099163 | 0.318 | 0.208 | 0.251 | 0.222 | 0.459 | 0.499 | 0.388 | 0.444 | 0.491 | 0.265 | 0.307 | 0.395 | 0.297 | 54.431 |
| MN099164 | 0.319 | 0.208 | 0.251 | 0.222 | 0.459 | 0.499 | 0.388 | 0.443 | 0.490 | 0.264 | 0.307 | 0.397 | 0.295 | 54.542 |
| MN099165 | 0.318 | 0.207 | 0.251 | 0.224 | 0.457 | 0.498 | 0.388 | 0.443 | 0.486 | 0.271 | 0.301 | 0.395 | 0.297 | 54.790 |
| MN099166 | 0.318 | 0.207 | 0.251 | 0.224 | 0.459 | 0.500 | 0.388 | 0.444 | 0.488 | 0.269 | 0.303 | 0.395 | 0.297 | 54.850 |
| MN099167 | 0.318 | 0.208 | 0.252 | 0.223 | 0.460 | 0.501 | 0.388 | 0.444 | 0.490 | 0.267 | 0.305 | 0.393 | 0.299 | 54.890 |
| MN099168 | 0.319 | 0.206 | 0.251 | 0.225 | 0.456 | 0.498 | 0.387 | 0.442 | 0.484 | 0.273 | 0.299 | 0.395 | 0.297 | 54.829 |
| MN099169 | 0.318 | 0.207 | 0.251 | 0.224 | 0.458 | 0.501 | 0.388 | 0.444 | 0.485 | 0.271 | 0.301 | 0.396 | 0.295 | 54.735 |
| MN099170 | 0.319 | 0.206 | 0.250 | 0.225 | 0.456 | 0.498 | 0.387 | 0.443 | 0.483 | 0.272 | 0.299 | 0.396 | 0.296 | 54.825 |
| MN564752 | 0.319 | 0.207 | 0.250 | 0.224 | 0.457 | 0.500 | 0.388 | 0.444 | 0.484 | 0.270 | 0.302 | 0.397 | 0.295 | 54.192 |
| NC_038964 | 0.317 | 0.206 | 0.253 | 0.224 | 0.459 | 0.496 | 0.384 | 0.440 | 0.496 | 0.265 | 0.305 | 0.387 | 0.309 | 55.171 |
| Average | 0.317 | 0.208 | 0.253 | 0.223 | 0.460 | 0.500 | 0.386 | 0.443 | 0.495 | 0.264 | 0.308 | 0.390 | 0.302 | 54.832 |
| SD | 0.001 | 0.001 | 0.001 | 0.001 | 0.002 | 0.002 | 0.002 | 0.001 | 0.007 | 0.006 | 0.004 | 0.005 | 0.006 | 0.254 |

Supplementary Table 3. The relative synonymous codon usage (RSCU) of APPV genotypes. Optimal codons are displayed in bold, and over-represented (RSCU>1.6) codons are marked in italics.

| Codon (AA) | Phylogroup I | Phylogroup II | Phylogroup III | All |
| --- | --- | --- | --- | --- |
| GCA(A) | **1.354±0.024** | **1.374±0.029** | **1.367±0.077** | **1.367±0.06** |
| GCC(A) | 1.24±0.026 | 1.303±0.036 | 1.362±0.097 | 1.328±0.087 |
| GCG(A) | 0.315±0.018 | 0.325±0.028 | 0.351±0.058 | 0.338±0.048 |
| GCU(A) | 1.091±0.024 | 0.997±0.041 | 0.92±0.087 | 0.966±0.092 |
| UGC(C) | 0.941±0.041 | **1.018±0.075** | **1.125±0.07** | **1.068±0.097** |
| UGU(C) | **1.059±0.041** | 0.982±0.075 | 0.875±0.07 | 0.932±0.097 |
| GAC(D) | **1.242±0.025** | **1.268±0.024** | **1.153±0.061** | **1.2±0.073** |
| GAU(D) | 0.758±0.025 | 0.732±0.024 | 0.847±0.061 | 0.8±0.073 |
| GAA(E) | **1.215±0.009** | **1.157±0.01** | **1.23±0.023** | **1.206±0.038** |
| GAG(E) | 0.785±0.009 | 0.843±0.01 | 0.77±0.023 | 0.794±0.038 |
| UUC(F) | **1.068±0.02** | **1.014±0.039** | **1.069±0.082** | **1.052±0.07** |
| UUU(F) | 0.932±0.02 | 0.986±0.039 | 0.931±0.082 | 0.948±0.07 |
| GGA(G) | 0.818±0.018 | 0.965±0.03 | 0.893±0.033 | 0.905±0.056 |
| GGC(G) | 1.061±0.032 | 0.799±0.025 | 0.914±0.057 | 0.899±0.094 |
| GGG(G) | **1.503±0.008** | **1.433±0.032** | **1.312±0.055** | **1.374±0.087** |
| GGU(G) | 0.619±0.028 | 0.802±0.027 | 0.881±0.035 | 0.823±0.093 |
| CAC(H) | 0.916±0.023 | **1.042±0.03** | 0.87±0.111 | 0.928±0.115 |
| CAU(H) | **1.084±0.023** | 0.958±0.03 | **1.13±0.111** | **1.072±0.115** |
| AUA(I) | **1.419±0.014** | **1.435±0.014** | **1.384±0.049** | **1.404±0.045** |
| AUC(I) | 0.87±0.018 | 0.867±0.021 | 0.903±0.052 | 0.888±0.045 |
| AUU(I) | 0.711±0.019 | 0.698±0.019 | 0.713±0.056 | 0.708±0.044 |
| AAA(K) | **1.231±0.011** | **1.072±0.013** | **1.178±0.045** | **1.153±0.066** |
| AAG(K) | 0.769±0.011 | 0.928±0.013 | 0.822±0.045 | 0.847±0.066 |
| CUA(L) | **1.211±0.042** | 1.205±0.029 | 1.192±0.102 | 1.199±0.08 |
| CUC(L) | 0.639±0.023 | 0.683±0.025 | 0.689±0.064 | 0.681±0.053 |
| CUG(L) | 1.108±0.033 | **1.391±0.023** | **1.45±0.073** | **1.387±0.127** |
| CUU(L) | 0.697±0.024 | 0.602±0.016 | 0.593±0.043 | 0.61±0.049 |
| UUA(L) | 1.199±0.037 | 1.072±0.025 | 1.034±0.082 | 1.067±0.084 |
| UUG(L) | 1.145±0.032 | 1.047±0.054 | 1.042±0.061 | 1.057±0.065 |
| AAC(N) | **1.085±0.036** | **1.021±0.028** | 0.917±0.061 | 0.971±0.082 |
| AAU(N) | 0.915±0.036 | 0.979±0.028 | **1.083±0.061** | **1.029±0.082** |
| CCA(P) | **1.5±0.023** | **1.566±0.032** | **1.431±0.078** | **1.481±0.087** |
| CCC(P) | 0.81±0.023 | 0.859±0.032 | 0.896±0.058 | 0.873±0.056 |
| CCG(P) | 0.727±0.018 | 0.576±0.047 | 0.584±0.07 | 0.601±0.077 |
| CCU(P) | 0.963±0.014 | 0.999±0.027 | 1.089±0.082 | 1.045±0.082 |
| CAA(Q) | **1.07±0.01** | **1.063±0.012** | **1.013±0.033** | **1.035±0.037** |
| CAG(Q) | 0.93±0.01 | 0.937±0.012 | 0.987±0.033 | 0.965±0.037 |
| AGA(R) | *2.084±0.058* | ***2.355±0.06*** | ***2.321±0.134*** | ***2.3±0.137*** |
| AGG(R) | ***2.465±0.045*** | *2.143±0.068* | *2.19±0.108* | *2.212±0.136* |
| CGA(R) | 0.409±0.052 | 0.37±0.042 | 0.413±0.075 | 0.399±0.066 |
| CGC(R) | 0.178±0.025 | 0.298±0.044 | 0.306±0.049 | 0.286±0.062 |
| CGG(R) | 0.606±0.022 | 0.436±0.03 | 0.469±0.093 | 0.477±0.089 |
| CGU(R) | 0.258±0.023 | 0.399±0.076 | 0.301±0.044 | 0.325±0.074 |
| AGC(S) | 1.172±0.036 | 1.173±0.073 | 1.148±0.076 | 1.159±0.071 |
| AGU(S) | 1.121±0.025 | **1.179±0.072** | 1.108±0.088 | 1.131±0.083 |
| UCA(S) | **1.214±0.022** | 1.166±0.076 | **1.424±0.089** | **1.318±0.146** |
| UCC(S) | 1.007±0.02 | 1.07±0.033 | 1.056±0.078 | 1.054±0.064 |
| UCG(S) | 0.563±0.039 | 0.358±0.059 | 0.343±0.092 | 0.377±0.106 |
| UCU(S) | 0.923±0.023 | 1.055±0.049 | 0.921±0.083 | 0.961±0.092 |
| ACA(U) | 1.151±0.02 | 1.124±0.037 | 1.124±0.052 | 1.128±0.045 |
| ACC(U) | **1.371±0.034** | **1.338±0.021** | **1.242±0.09** | **1.288±0.088** |
| ACG(U) | 0.438±0.013 | 0.401±0.023 | 0.428±0.073 | 0.421±0.058 |
| ACU(U) | 1.041±0.036 | 1.136±0.015 | 1.206±0.086 | 1.163±0.087 |
| GUA(V) | 1.059±0.018 | 0.991±0.05 | 1.066±0.067 | 1.042±0.066 |
| GUC(V) | 1.117±0.036 | 1.146±0.027 | 1.18±0.066 | 1.161±0.057 |
| GUG(V) | **1.429±0.02** | **1.374±0.028** | **1.324±0.066** | **1.353±0.064** |
| GUU(V) | 0.394±0.021 | 0.49±0.037 | 0.43±0.054 | 0.443±0.056 |
| UAC(Y) | **1.063±0.031** | **1.026±0.027** | 0.935±0.06 | 0.979±0.071 |
| UAU(Y) | 0.937±0.031 | 0.974±0.027 | **1.065±0.06** | **1.021±0.071** |

Supplementary Table 4. Relative dinucleotide frequencies of APPV phylogroups. The over-represented (>1.23) dinucleotides are displayed in bold, and the under-represented (<0.78) dinucleotides are marked in italics.

| Dinucleotides | Phylogroup I | Phylogroup II | Phylogroup III | All |
| --- | --- | --- | --- | --- |
| AA | 1.058±0.002 | 1.026±0.003 | 1.06±0.008 | 1.05±0.017 |
| AC | 0.97±0.007 | 0.957±0.006 | 0.929±0.016 | 0.943±0.02 |
| AG | 1.01±0.002 | 1.044±0.004 | 1.01±0.008 | 1.021±0.017 |
| AT | 0.934±0.004 | 0.953±0.005 | 0.967±0.013 | 0.958±0.016 |
| CA | 1.075±0.005 | 1.093±0.004 | 1.053±0.009 | 1.068±0.02 |
| CC | **1.288±0.006** | **1.318±0.01** | **1.315±0.018** | **1.312±0.018** |
| CG | *0.495±0.004* | *0.437±0.013* | *0.485±0.018* | *0.472±0.028* |
| CT | 1.2±0.006 | 1.215±0.009 | 1.214±0.022 | 1.213±0.018 |
| GA | 0.964±0.004 | 1.006±0.004 | 0.987±0.01 | 0.99±0.016 |
| GC | 0.884±0.01 | 0.845±0.005 | 0.874±0.017 | 0.866±0.02 |
| GG | 1.223±0.002 | 1.189±0.005 | 1.21±0.014 | 1.205±0.016 |
| GT | 0.904±0.007 | 0.92±0.003 | 0.901±0.011 | 0.907±0.012 |
| TA | 0.887±0.005 | 0.868±0.006 | 0.88±0.013 | 0.877±0.012 |
| TC | 0.908±0.012 | 0.942±0.009 | 0.951±0.018 | 0.943±0.021 |
| TG | 1.203±0.003 | **1.249±0.01** | 1.227±0.022 | **1.23±0.022** |
| TT | 1.017±0.007 | 0.958±0.009 | 0.96±0.021 | 0.967±0.026 |

Supplementary Table 5. Comparison of genetic distances between phylogroups.

| Group1 | Group2 | Genetic distances | Min | Q1 | Median | Q3 | Max |
| --- | --- | --- | --- | --- | --- | --- | --- |
| Phylogroup 1 | Phylogroup 2 | 0.2451±0.0002 | 0.2413 | 0.2438 | 0.2452 | 0.2463 | 0.2502 |
| Phylogroup 1 | Phylogroup 3 | 0.2350±0.0003 | 0.2264 | 0.2324 | 0.2356 | 0.2378 | 0.2436 |
| Phylogroup 2 | Phylogroup 3 | 0.2063±0.0001 | 0.2001 | 0.2039 | 0.2062 | 0.2084 | 0.2134 |
