# Supplementary material for: Phylogenetic and codon usage analysis of atypical porcine pestivirus (APPV)
Source: Virulence. 2020 Jul 29;11(1):916–26. doi: 10.1080/21505594.2020.1790282 (PMC7549985; doi:10.1080/21505594.2020.1790282)
Supplement: Supplemental Material [file KVIR_A_1790282_SM8595.docx]

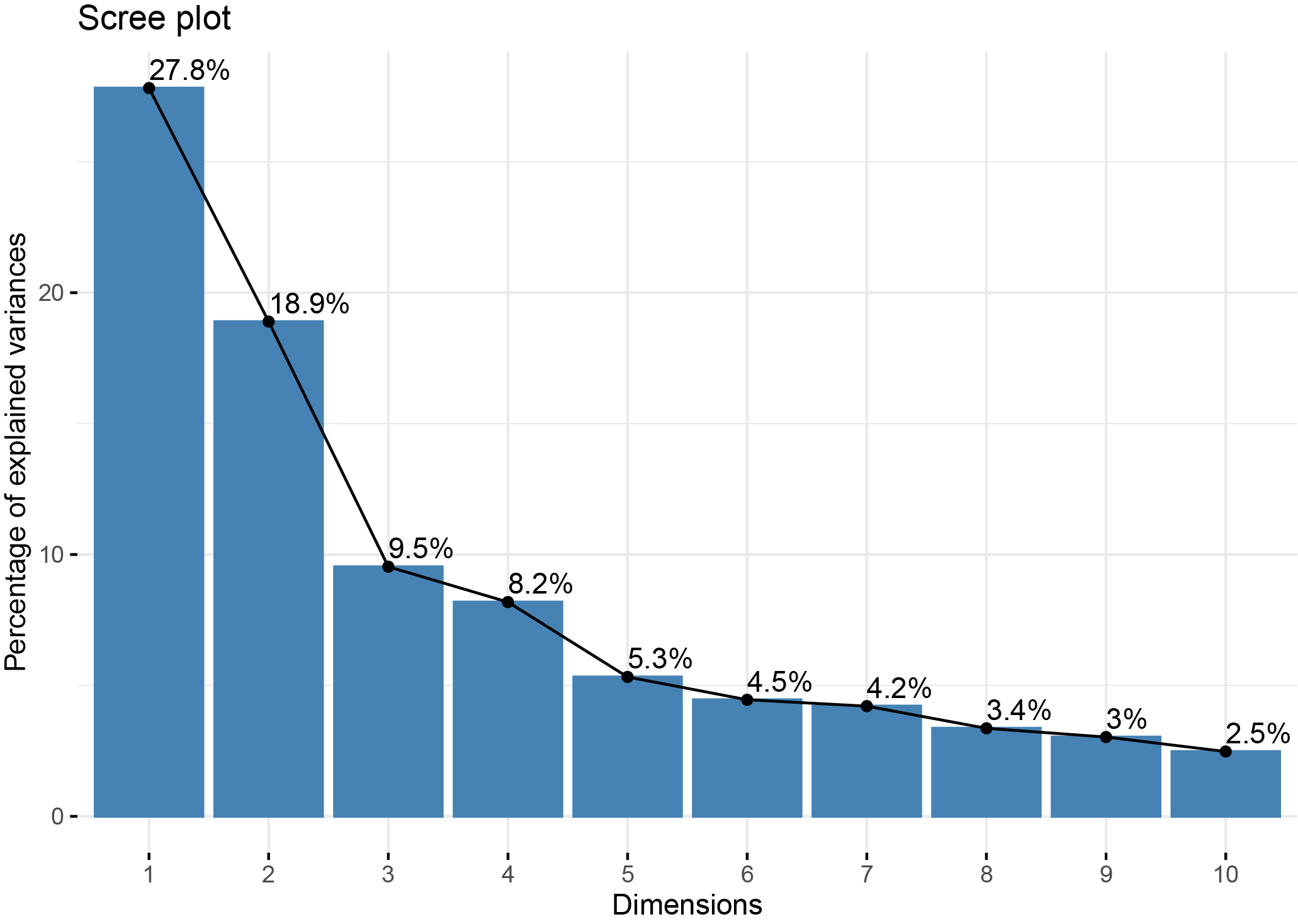


**Supplementary Figure 1. PCA scree plot for the percentage of explained variances.** Scree plot extracts and visualizes eigenvalues (variances) from the RSCU values, and shows the proportion of total variance for each principal component (dimension) in descending order of magnitude.


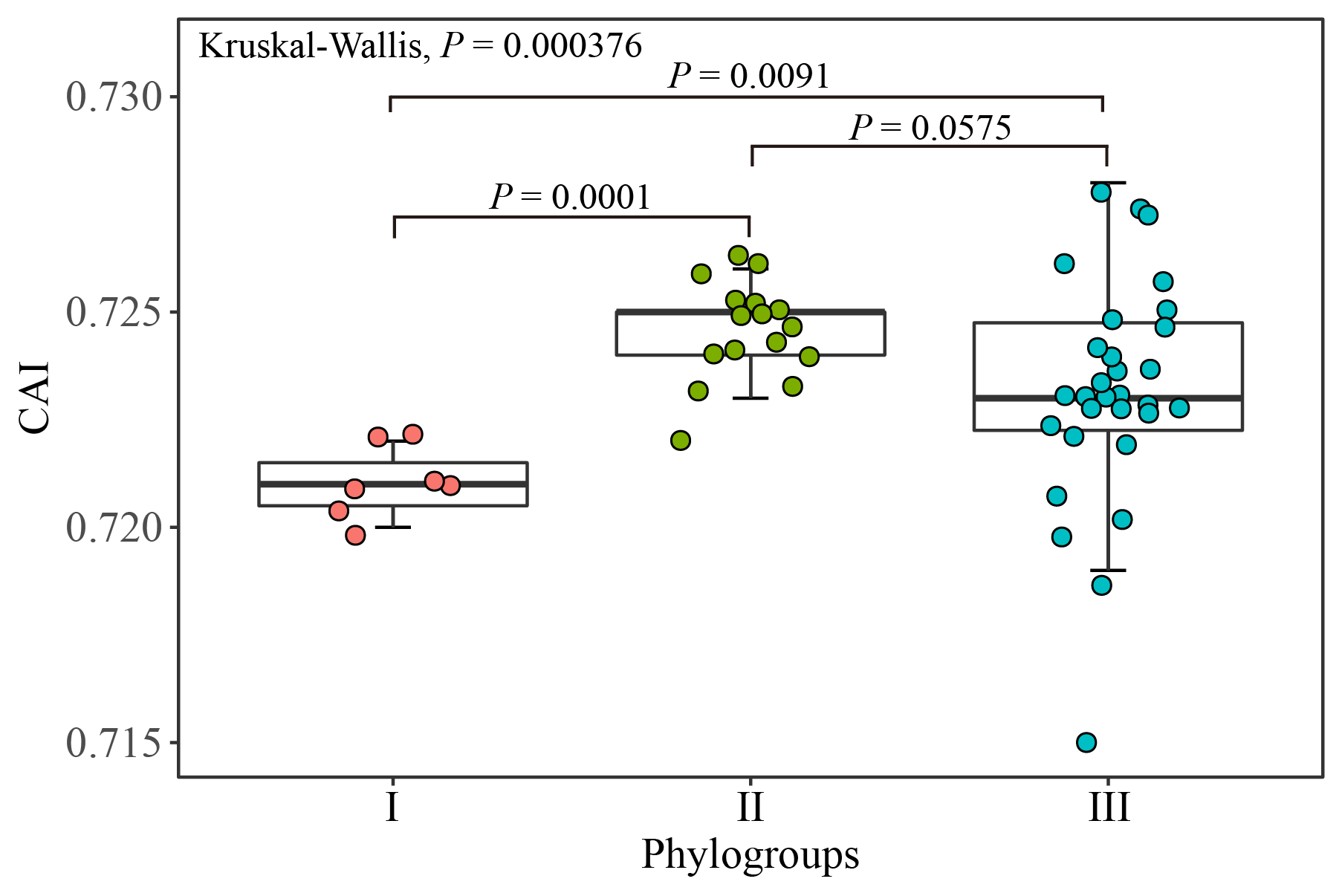


**Supplementary Figure 2. CAI of the phylogroups of APPV.** The abscissa denotes the phylogroups and the ordinate denotes the CAI values. Phylogroups I, II, and III are represented in orange, green, and blue, respectively.


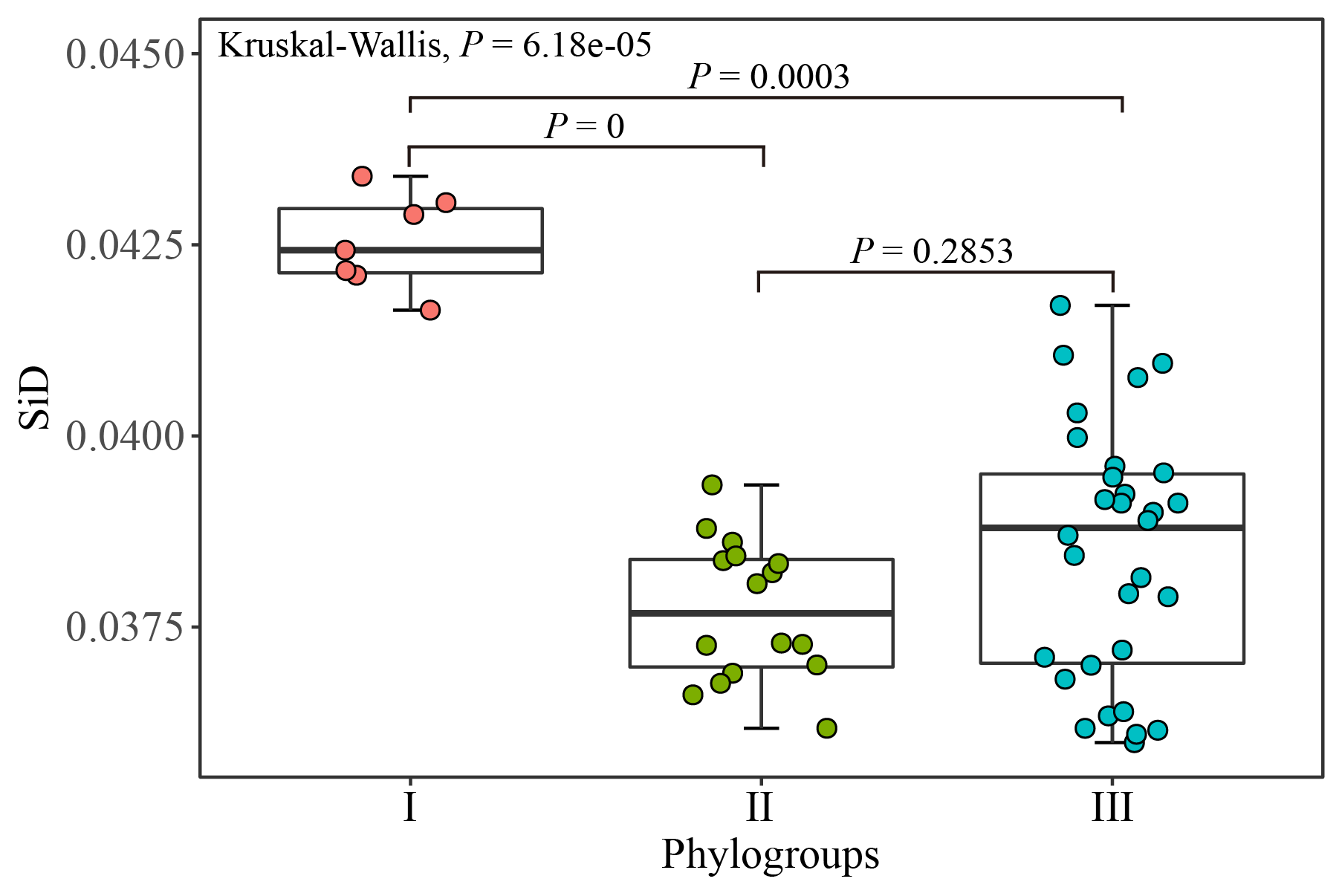


**Supplementary Figure 3. SiD of the phylogroups of APPV.** The abscissa denotes the phylogroups and the ordinate denotes the SiD values. Phylogroups I, II, and III are represented in orange, green, and blue, respectively.


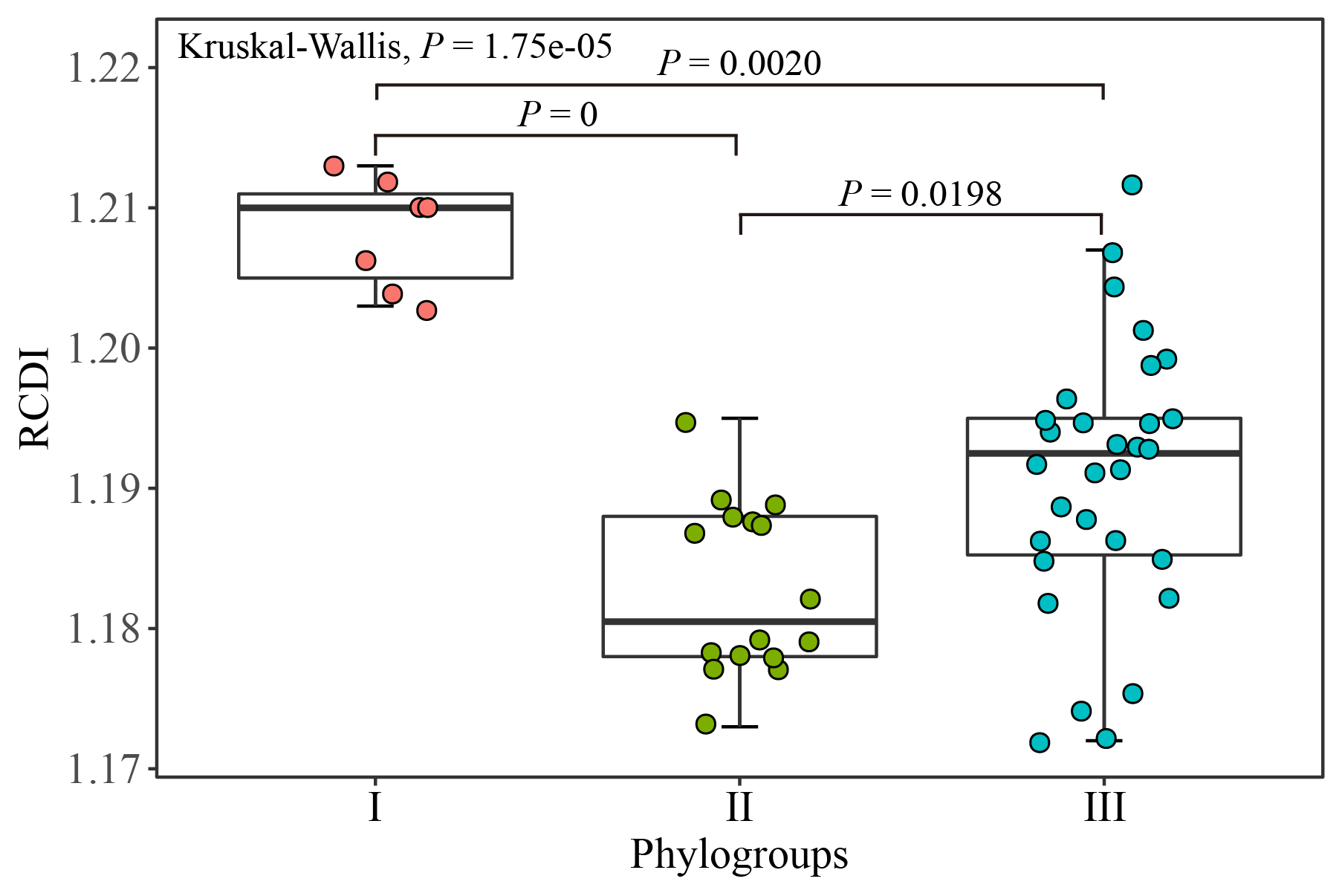


**Supplementary Figure 4. RCDI of the phylogroups of APPV.** The abscissa denotes the phylogroups and the ordinate denotes the RCDI values. Phylogroups I, II, and III are represented in orange, green, and blue, respectively.
